# Association between the Use of Proton Pump Inhibitors and Cardiovascular Diseases: A Nested Case-Control Study Using a National Health Screening Cohort

**DOI:** 10.3390/biomedicines12010170

**Published:** 2024-01-12

**Authors:** Sarang Jang, Hyo Geun Choi, Mi Jung Kwon, Ji Hee Kim, Joo-Hee Kim, Yoonjoong Kim, So Young Kim

**Affiliations:** 1 Department of Public Health, Sahmyook University, Seoul 01795, Republic of Korea; jangsarang@gmail.com; 2 Mdanalytics, Seoul 06349, Republic of Korea; pupen@naver.com; 3 Suseoseoulent Clinic, Seoul 06349, Republic of Korea; 4 Department of Pathology, Hallym University Sacred Heart Hospital, Hallym University College of Medicine, Anyang 14068, Republic of Korea; mulank99@hallym.or.kr; 5 Department of Neurosurgery, Hallym University Sacred Heart Hospital, Hallym University College of Medicine, Anyang 14068, Republic of Korea; kimjihee@hallym.or.kr; 6 Department of Medicine, Hallym University Sacred Heart Hospital, Hallym University College of Medicine, Anyang 14068, Republic of Korea; luxjhee@hallym.or.kr; 7 Department of Otorhinolaryngology-Head and Neck Surgery, Chungbuk National University Hospital, Cheongju 28644, Republic of Korea; mdykim@gmail.com; 8 Department of Otorhinolaryngology-Head & Neck Surgery, CHA Bundang Medical Center, CHA University, Seongnam 13496, Republic of Korea

**Keywords:** brain neoplasms, Hydroxymethylglutaryl-CoA reductase inhibitors, cohort studies, case-control studies, epidemiology

## Abstract

We investigated the association of proton pump inhibitor (PPI) use with the risk of stroke and ischemic heart disease (IHD). The Korean National Health Insurance Service-Health Screening cohort from 2002 to 2003, the participants of which were followed up until 2019, was used. In study I, 45,905 participants who were diagnosed with stroke were matched with 91,810 control I participants. The history of PPI medication was examined. In study II, 40,928 participants who were diagnosed with IHD were matched with 81,856 control II participants. In both study I and study II, the previous history of PPI medication was examined. A propensity score overlap-weighted multivariable logistic regression analysis was conducted to estimate the overlap-weighted odds ratios (ORs) of PPI use for stroke (study I) and IHD (study II). Current PPI use was linked with higher odds for stroke in study I. The odds for stroke were higher in groups with a longer duration of PPI use (OR = 0.96 [95% CI = 0.92–1.00] < 1.55 [1.50–1.61] < 1.62 [1.57–1.68] for < 30 days, 30 to 180 days, and ≥180 days of PPI use). Previous PPI use was linked with higher odds for IHD in study II. The odds for stroke were higher in groups with a longer duration of PPI use (OR = 1.13 [95% CI = 1.08–1.18] < 2.12 [2.04–2.21] < 2.60 [2.51–2.69] for <30 days, 30 to 180 days, and ≥180 days of PPI use). Current PPI medication is associated with a high risk of stroke and IHD. A longer duration of PPI medication was related to a higher risk of stroke and IHD. However, a prior history of PPI medication was not linked with a high risk of stroke or IHD.

## 1. Introduction

Proton pump inhibitors (PPIs) are one of the most commonly used medicines with widespread indications for gastroesophageal reflux disease (GERD), peptic ulcers, and other indications to repress gastric acid-related disorders. PPIs act by inhibiting 70–80% of the active potassium pumps that reside in the apical membrane of gastric parietal cells, diminishing acid synthesis from the stomach [1]. Novel gastric pumps are synthesized continuously, which takes approximately 36–96 h. Thus, the effect of a PPI on gastric acidity lasts approximately 12 h when it is prescribed as a one-daily regimen. Thus, PPIs have been considered to have fewer side effects and demand repetitive medication. Indeed, PPIs showed an excellent safety profile, and less than 1% of patients complained of minor adverse symptoms such as headache, nausea, and abdominal discomfort [2]. The effectiveness for acid control and safety of drugs have paved the way to the wide use of PPIs. However, the expanding indication for the preventive management of gastroduodenal ulcers in patients without any risks, for instance, in nonintensive care units, steroid therapy alone, and antiplatelet or anticoagulant therapy without any risk for gastric ulcers, has imposed concerns of inadvertent complications from PPI overuse [3].

Many previous studies have examined the potential adverse effects of PPIs. The prolonged suppression of acid can lead to an increased gastric pH, hypochlorhydria, and achlorhydria, which increase the risk of congenital malformations in pregnant women and accelerate the metaplastic transformation of gastric polyps, carcinoids, and carcinomas [4].

In addition, PPIs were supposed to suppress the renal tubular proton pump, in addition to gastric pumps, which increase the risk of chronic renal disease [5]. Other disease risks, such as osteoporosis-related fractures, dementia, and liver disease, have also been reported by diverse researchers. However, there has also been some criticism of the noncausal association between PPI use and its proposed adverse effects [6].

There has been supporting evidence on the additional risk of cardiovascular diseases associated with PPI use [7]. PPIs are known to be a competitive inhibitor of CYP2C19, which metabolizes the clopidogrel prodrug to its active form [8]. Thus, the combinational use of PPIs with antiplatelets was suggested to elevate the risk of cardiac complications [9]. In addition, by inhibiting dimethylarginine dimethylaminohydrolase (DDAH), PPIs have been suggested to impair endothelial function [10]. However, the impact of PPIs on cardiovascular diseases has been controversial [11,12]. A meta-analysis estimated that there was no relationship between PPI use and cardiovascular disease in randomized controlled studies (risk ratio = 0.89, 95% confidence intervals [CI] = 0.34–2.33, *p* = 0.85).

We aimed to investigate the risk of cardiovascular disease, especially stroke and ischemic heart disease (IHD), related to PPI use. The incidence of stroke and IHD was counted following PPI medication to estimate the effect of PPIs on the new incidence of these diseases. The comorbidities and demographic and lifestyle factors were collected and matching and adjusting were conducted to alleviate the potential confounding effects from these variables.

## 2. Materials and Methods

### 2.1. Exposure (Proton Pump Inhibitors)

Proton pump inhibitor (PPI) use was defined as the prescription and duration during the 1 year before the index date. In this study, PPI duration was categorized into four categories: non-user, <30 days, 30 to 180 days, and ≥180 days.

### 2.2. Outcome (Cardiovascular Diseases; CVDs)

Stroke and IHD were selected based on ICD-10 codes (I60-I69 for stroke and I20-I25 for IHD). We only included participants who were hospitalized ≥2 days or who died because of each disease, as described in our previous studies [13,14].

### 2.3. Participant Selection

The detailed description of the Korean National Health Insurance Service-Health Screening (NHIS-HealS) cohort data (2002–2003, follow-up until 2019) was described elsewhere [15,16,17].

#### 2.3.1. Study I

**Figure 1:**
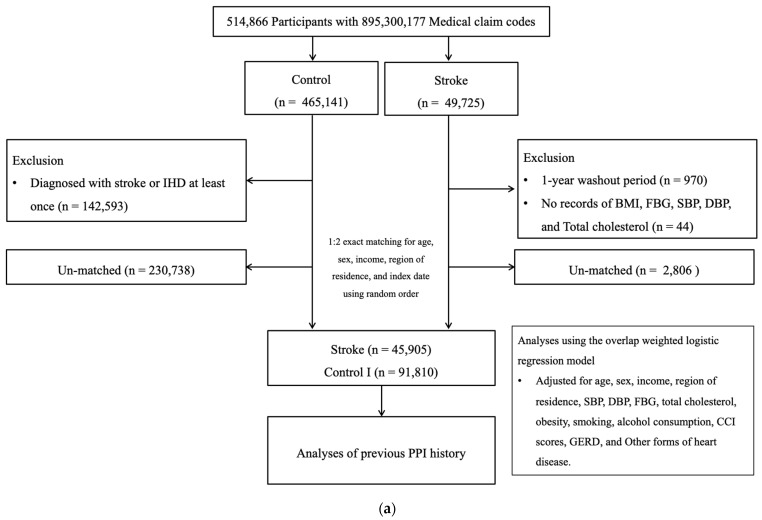

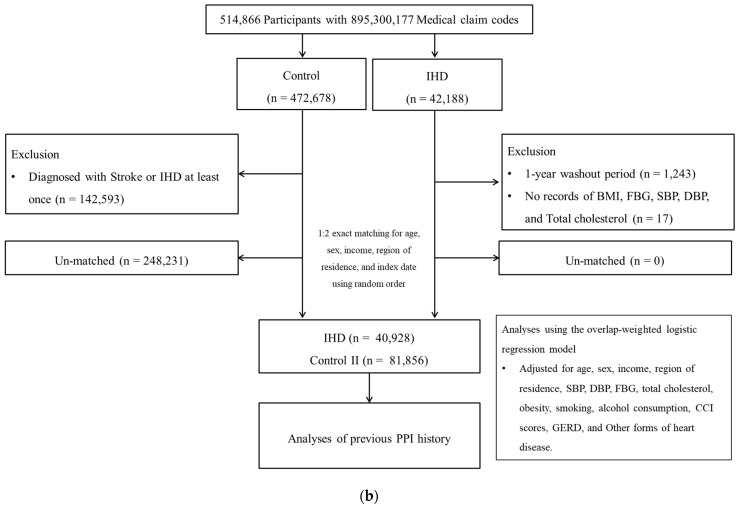
(**a**) A schematic illustration of the participant selection process that was used in the present study I. Of a total of 514,866 participants, 45,905 stroke participants were matched with 91,810 control participants for age, sex, income, and region of residence. (**b**) A schematic illustration of the participant selection process that was used in the present study II. Of a total of 514,866 participants, 40,928 IHD participants were matched with 81,856 control II participants for age, sex, income, and region of residence.

Among 514,866 participants (2002–2019), the participants with stroke were identified (n = 49,725). The participants without history of stroke were randomly selected to collect the control I participants (n = 465,141). The participants who were diagnosed with stroke in 2002 were removed from the study population (n = 970). Stroke participants who did not have records of BMI, blood pressure, fasting blood glucose, and total cholesterol (n = 44) were excluded. Control I participants who were diagnosed with stroke or IHD at least once (n = 142,593) were excluded. The participants with stroke were 1:2 matched with control I participants. The date of initial diagnosis of stroke was defined as the index date. As a result, 2806 stroke participants and 230,738 control I participants were excluded. Ultimately, 45,905 stroke participants were 1:2 matched with 91,810 control I participants (Figure 1a).

#### 2.3.2. Study II

IHD participants were selected from 514,866 participants with 895,300,177 medical claim codes from 2002 to 2019 (n = 42,188). The control II group was included if participants were not defined as having IHD from 2002 to 2019 (n = 472,678). To select IHD participants who were diagnosed for the first time, IHD participants diagnosed in 2002 were excluded (washout periods, n = 1243). IHD participants who did not have records of BMI, blood pressure, fasting blood glucose, and total cholesterol (n = 17) were excluded. Control II participants who were diagnosed with stroke or IHD at least once (n = 142,593) were excluded. IHD participants were 1:2 matched with control II participants. The date of initial diagnosis of IHD was defined as the index date. To sum up, 40,928 IHD participants were 1:2 matched with 81,856 control II participants (Figure 1b).

### 2.4. Covariates

Age groups were divided into 5-year intervals: 40–44, 45–49…, and 85+ years old (total of 10 age groups). The level of income, region of residence, histories of tobacco smoking and alcohol consumption, obesity group based on BMI (body mass index, kg/m^2^), systolic blood pressure (SBP, mmHg), diastolic blood pressure (DBP, mmHg), fasting blood glucose (FBG, mg/dL), and total cholesterol (mg/dL) were collected and adjusted in the analyses [18,19]. 

The Charlson Comorbidity Index (CCI) was counted, except for cerebrovascular disease, congestive heart failure, and acute myocardial infarction, and adjusted in this study.

The number of patients diagnosed with GERD (ICD-10 code: K21, treated ≥2 times and prescribed a PPI for ≥2 weeks) for 1 year prior to the index date was additionally assessed.

Regarding CVDs, other forms of heart disease (ICD-10 codes: I30-I52) were further evaluated.

### 2.5. Statistical Analyses

We conducted propensity score overlap weighting analyses to reflect the covariate balance and effective sample size [20,21]. The propensity score (PS) was applied and overlap weighting was calculated [22,23]. The variables of study and control groups were compared using the standardized difference.

To analyze the overlap-weighted odds ratios (ORs) of prescription dates of proton pump inhibitors for stroke and IHD, a propensity score overlap-weighted multivariable logistic regression analysis was used. In these analyses, crude and overlap-weighted models were used.

The 95% confidence interval (CI) was calculated. Subgroup analyses were conducted.

Two-tailed analyses were performed, and significance was defined as *p* values less than 0.05. SAS version 9.4 (SAS Institute Inc., Cary, NC, USA) was used for statistical analyses.

## 3. Results

A total of 12.36% (5197/45,905) of stroke patients and 20.09% (16,893/91,810) of control I participants were non-PPI users (Table S1). There were differences in the distributions of obesity groups, smoking status, alcohol consumption, blood pressure, fasting blood glucose, total cholesterol, CCI score, history of GERD, and other forms of heart disease between the stroke and control I groups. Thus, all these variables were included in the PS overlap weight adjustment. After the PS overlap weight adjustment, 13.21% of stroke patients and 18.00% of control I participants were non-PPI users (sd = 0.22, Table 1). 

The ≥180 days of PPI use was associated with 2.13-fold higher odds for stroke in the crude model (95% CI = 2.06–2.21, *p* < 0.001, Table 2). In the overlap-weighted model, the odds for stroke were 1.62-fold higher in the ≥180 days of PPI users (95% CI = 1.57–1.68, *p* < 0.001). The odds for stroke were higher when the duration of PPI use was longer (OR = 0.96 [95% CI = 0.92–1.00] < 1.55 [1.50–1.61] < 1.62 [1.57–1.68] for <30 days, 30 to 180 days, and ≥180 days of PPI use). 

**Figure 2:**
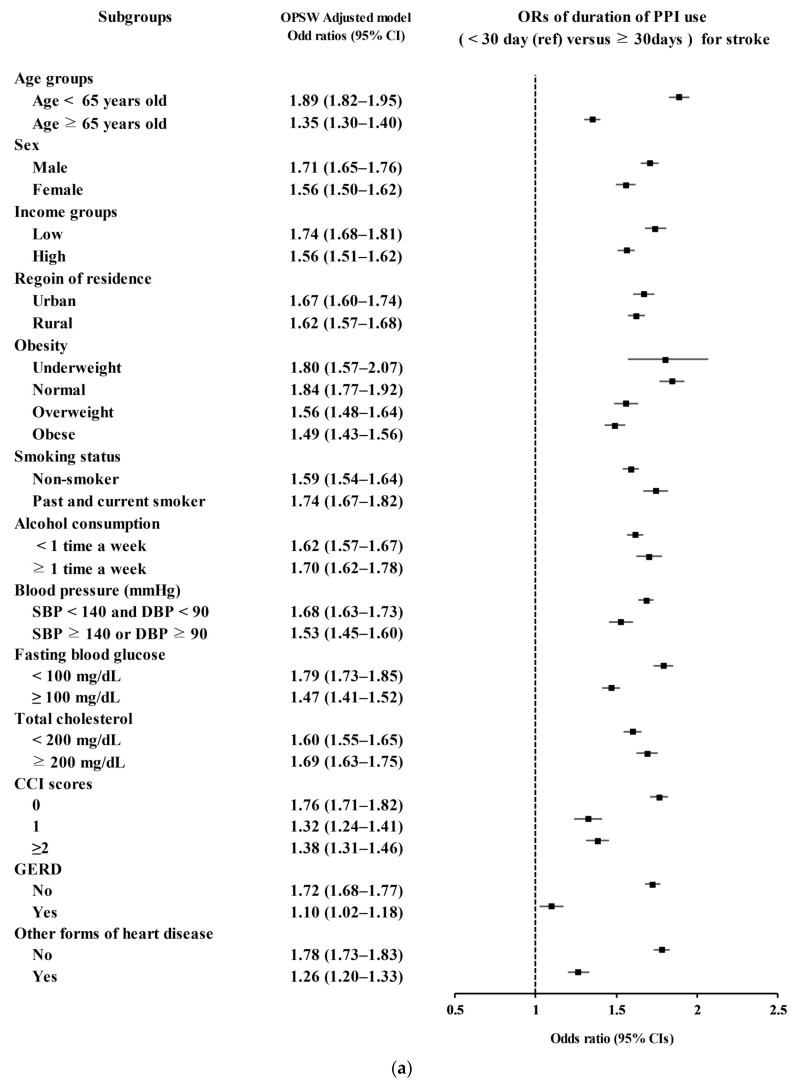

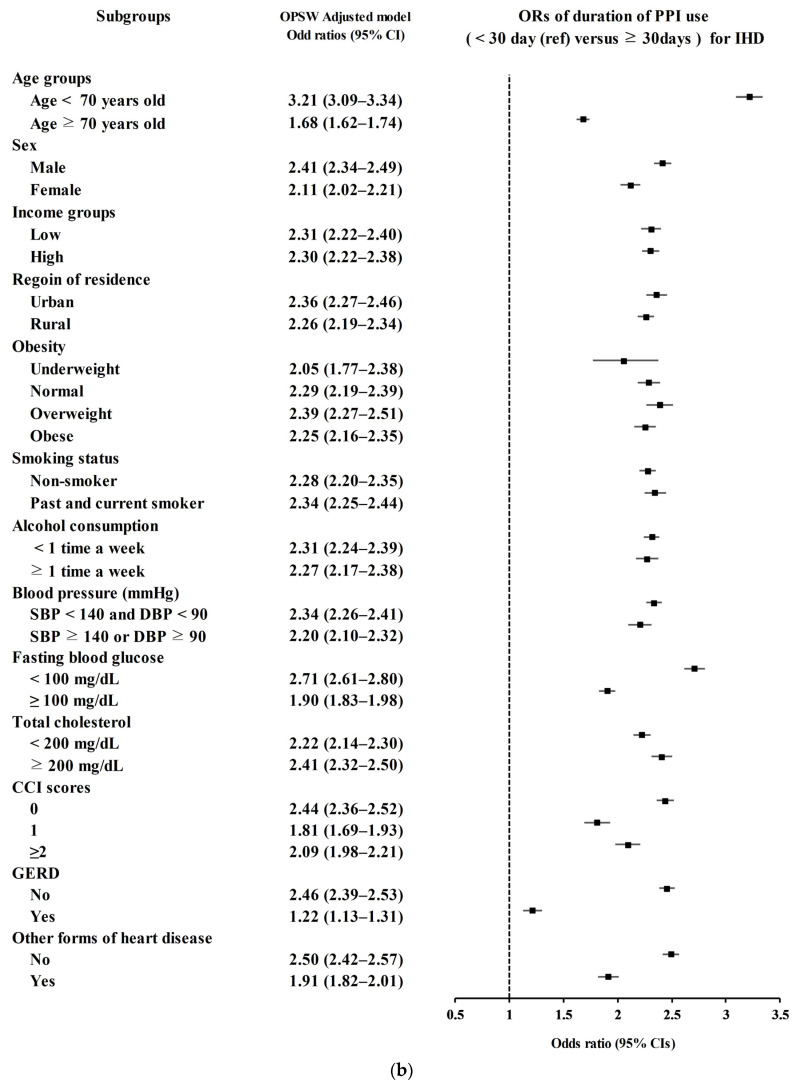
(**a**) Overlap propensity score-weighted (OPSW) odds ratios (OR) of PPI for stroke. (**b**) Overlap propensity score-weighted (OPSW) odds ratios (OR) of PPI for ischemic heart disease (IHD).

Secondary analyses according to age, sex, income, region of residence, obesity, smoking status, alcohol consumption, blood pressure, fasting blood glucose, total cholesterol, CCI scores, GERD, and other forms of heart disease demonstrated consistently higher odds for stroke in PPI users (Figure 2a).

In total, 9.27% (3795/40,928) of IHD patients and 20.67% (16,917/81,856) of control II participants were non-PPI users (sd = 0.52, Table S1). There were differences in the distributions of obesity groups, smoking status, alcohol consumption, blood pressure, fasting blood glucose, total cholesterol, CCI score, history of GERD, and other forms of heart disease between the IHD and control II groups. Thus, all these variables were included in the PS overlap weight adjustment. After the PS overlap weight adjustment, 10.05% of IHD patients and 18.57% of control II participants were non-PPI users (sd = 0.37, Table 1). 

The ≥ 180 days of PPI use was associated with 3.41-fold higher odds for IHD in the crude model (95% CI = 3.28–3.55, *p* < 0.001, Table 3). In the overlap-weighted model, the odds for IHD were 2.60-fold higher in the ≥180 days of PPI users (95% CI = 2.51–2.69, *p* < 0.001). The odds for IHD were higher when the duration of PPI use was longer (OR = 1.13 [95% CI = 1.08–1.18] < 2.12 [2.04–2.21] < 2.60 [2.51–2.69] for <30 days, 30 to 180 days, and ≥180 days of PPI use). 

Secondary analyses according to age, sex, income, region of residence, obesity, smoking status, alcohol consumption, blood pressure, fasting blood glucose, total cholesterol, CCI scores, GERD, and other forms of heart disease demonstrated consistently higher odds for IHD in PPI users (Figure 2b). 

## 4. Discussion

PPI use was related to an increased incidence of stroke and IHD in this study. The risks of stroke and IHC were predicted to be higher in patients with a longer duration of PPI use.

Therefore, it can be presumed that PPI use can be associated with an increased incidence of stroke and IHD in past PPI users with a dose-response relationship. Past PPI users may show an increased incidence of stroke and IHD because they may have higher risk characteristics, such as chest tightness, which can lead to mistakenly identifying the symptoms of IHD as gastric reflux symptoms. In summary, the potential impacts of PPIs on stroke and IHD can be mixed with the possible harmful effects of PPIs and misusage of PPIs in inappropriate indications. Although the odds of PPI use for the incidence of stroke and IHD were high in the current study, we cannot conclude the direct adverse effects of PPIs on the cardiovascular system.

The adverse effects of PPIs on the cardiovascular system have been actively discussed by many researchers with some conflicting results [24]. A number of previous studies have suggested a high risk of stroke and IHD in PPI users. A review study using ChatGPT estimated that most observational studies indicated a positive relationship between PPI use and major cardiovascular events [24]. A nationwide study in Denmark demonstrated a 1.13 times higher risk of ischemic stroke in PPI users (95% CI = 1.09–1.19) [25]. They also reported an increased risk of myocardial infarction in PPI users (hazard ratio = 1.31, 95% CI = 1.23–1.39). The increased risk of stroke and myocardial infarction was higher in long-term and high-dose PPI users. Moreover, a prospective study using the UK Biobank estimated that the risk of stroke was 1.16-fold higher in PPI users than in non-users (95% CI = 1.06–1.27) [26]. They also conducted a meta-analysis that consolidated their finding with a 1.22-fold higher risk of stroke (95% CI = 1.00–1.50). However, a self-controlled case series study reported no increased risk of myocardial infarction in short-term PPI users compared to H2 receptor antagonist users [27]. They suggested potential bias, such as confounding effects and protopathic bias, in studies on the risk of PPIs.

PPIs can exert negative effects on the cardiovascular system by a number of plausible mechanisms. PPIs were suggested to impair endothelial function by inhibiting endothelial nitric oxide synthase and the absorption of vitamin C and B12, and increasing the risk of metabolic syndrome. PPIs are known to interfere with DDAH activity. The decreased DDAH activity increases the plasma level of asymmetric dimethylarginine (ADMA), which competitively inhibits nitric oxide (NO) synthesis [10]. Because NO is a crucial vasoprotective molecule that represses cell proliferation, platelet aggregation, and the interaction between endothelial cells and leukocytes, reduced NO synthesis can result in cardiovascular compromise [10]. In addition, PPIs were suggested to induce vitamin B12 deficiency by increasing homocysteine levels and elevating ADMA levels [28]. Moreover, it was reported that PPIs can increase the risk of metabolic syndrome and diabetes [29,30].

However, short-term PPI use was less linked with an elevated incidence of stroke or IHD in the present study. It can be supposed that the impact of PPI on stroke and IHD may not last for a long time. The half-life of PPI elimination was estimated to be approximately one hour [31]. In an animal study, the half-life for the recovery of proton pump activity following PPI use was predicted to be approximately 15 h after PPI use [31]. In addition, the symptoms of GERD can be similar or indistinguishable from those of cardiovascular disease. The burning sensation in the epigastric area and chest pain are common extraesophageal symptoms in patients with GERD [32]. Thus, patients with subclinical or mild symptoms of cardiovascular disease can be prescribed PPIs depending on their symptoms and delay the diagnosis of cardiovascular diseases, which can result in protopathic bias [33]. Moreover, PPIs are often prescribed along with other medicines, such as corticosteroids, anticoagulants, and nonsteroidal anti-inflammatory agents, to prevent medication-associated ulcers [34]. Thus, the high comorbidities and concurrent medication in high-risk patients for stroke and IHD can increase the rate of PPI use in these populations. Although we adjusted a considerable number of variables in the analyses, this possible reverse causality cannot be completely excluded.

This study was based on a large, nationwide cohort, through which we can achieve a large control population to alleviate the risk of selection bias. Because all Koreans must register with the national health insurance system, which is regularly monitored by statisticians hired by the Korean government, there was little concern about missing or duplicated data. However, because the current study was based on health claim codes, undiagnosed or misdiagnosed participants can be missed in the analysis. In addition, the accuracy of the diagnosis of stroke and IHD was objective and reliable because it was determined by physicians, and the severity and management of diseases were heterogeneous in this study. For PPI use, this study used prescription data. Thus, the compliance of patients with PPI prescriptions can influence the analytic results of the present study. Moreover, concurrent medication including aspirin was not considered in this study. Although this study investigated the incidence of stroke and IHD after PPI medication, the temporal relation between PPI use and stroke or IHD cannot be concluded. Because the study population of the current study was Korean, the results may be different in other ethnic or regional populations.

## 5. Conclusions

Previous PPI use was related to a higher risk of stroke and IHD. A longer duration of PPI use was associated with a higher risk of stroke and IHD than short-term PPI use. 

## Figures and Tables

**Table 1:** General characteristics of participants after propensity score overlap weighting adjustment.

Characteristics	After PS Overlap Weighting Adjustment			After PS Overlap Weighting Adjustment		
	Stroke	Control I	Standardized Difference	IHD	Control II	Standardized Difference
Age (%)			0.00			0.00
40–44	43 (0.16)	43 (0.16)		140 (0.59)	140 (0.59)	
45–49	553 (2.13)	553 (2.13)		871 (3.68)	871 (3.68)	
50–54	1621 (6.24)	1621 (6.24)		1945 (8.23)	1945 (8.23)	
55–59	3084 (11.87)	3084 (11.87)		3409 (14.41)	3409 (14.41)	
60–64	3630 (13.97)	3630 (13.97)		3884 (16.42)	3884 (16.42)	
65–69	4226 (16.26)	4226 (16.26)		4094 (17.31)	4094 (17.31)	
70–74	4878 (18.77)	4878 (18.77)		3795 (16.05)	3795 (16.05)	
75–79	4893 (18.83)	4893 (18.83)		3000 (12.69)	3000 (12.69)	
80–84	2843 (10.94)	2843 (10.94)		1742 (7.37)	1742 (7.37)	
85+	216 (0.83)	216 (0.83)		770 (3.26)	770 (3.26)	
Sex (%)			0.00			0.00
Male	14,005 (53.90)	14,005 (53.90)		14,538 (61.47)	14,538 (61.47)	
Female	11,979 (46.10)	11,979 (46.10)		9113 (38.53)	9113 (38.53)	
Income (%)			0.00			0.00
1 (lowest)	4718 (18.16)	4718 (18.16)		4009 (16.95)	4009 (16.95)	
2	3211 (12.36)	3211 (12.36)		2848 (12.04)	2848 (12.04)	
3	3976 (15.30)	3976 (15.30)		3517 (14.87)	3517 (14.87)	
4	5480 (21.09)	5480 (21.09)		4922 (20.81)	4922 (20.81)	
5 (highest)	8600 (33.10)	8600 (33.10)		8356 (35.33)	8356 (35.33)	
Region of residence (%)			0.00			0.00
Urban	9945 (38.27)	9945 (38.27)		9917 (41.93)	9917 (41.93)	
Rural	16,039 (61.73)	16,039 (61.73)		13,734 (58.07)	13,734 (58.07)	
Obesity ^†^ (%)			0.00			0.00
Underweight	883 (3.40)	883 (3.40)		662 (2.80)	662 (2.80)	
Normal	9023 (34.72)	9023 (34.72)		7481 (31.63)	7481 (31.63)	
Overweight	6865 (26.42)	6865 (26.42)		6426 (27.17)	6426 (27.17)	
Obese I	8366 (32.20)	8366 (32.20)		8252 (34.89)	8252 (34.89)	
Obese II	847 (3.26)	847 (3.26)		830 (3.51)	830 (3.51)	
Smoking status (%)			0.00			0.00
Non-smoker	17,625 (67.83)	17,625 (67.83)		14,996 (63.40)	14,996 (63.40)	
Past smoker	2132 (8.21)	2132 (8.21)		2349 (9.93)	2349 (9.93)	
Current smoker	6226 (23.96)	6226 (23.96)		6307 (26.67)	6307 (26.67)	
Alcohol consumption (%)			0.00			0.00
<1 time a week	19,021 (73.20)	19,021 (73.20)		17,040 (72.05)	17,040 (72.05)	
≥1 time a week	6963 (26.80)	6963 (26.80)		6611 (27.95)	6611 (27.95)	
SBP (Mean, SD)	130.85 (13.71)	130.85 (9.59)	0.00	130.12 (12.97)	130.12 (9.24)	0.00
DBP (Mean, SD)	79.39 (8.61)	79.39 (5.96)	0.00	79.24 (8.25)	79.24 (5.76)	0.00
FBG (Mean, SD)	105.74 (25.97)	105.74 (19.58)	0.00	106.02 (25.14)	106.02 (19.84)	0.00
Total cholesterol (Mean, SD)	197.50 (31.58)	197.50 (22.27)	0.00	199.03 (31.41)	199.03 (20.87)	0.00
CCI score (Mean, SD)	1.36 (1.29)	1.36 (1.14)	0.00	1.21 (1.28)	1.21 (1.01)	0.00
GERD for 1 year before index date (Mean, SD)	0.62 (1.57)	0.62 (1.26)	0.00	0.66 (1.41)	0.66 (1.41)	0.00
Other forms of heart disease (n, %)	8151 (31.37)	8151 (31.37)	0.00	8769 (37.08)	8769 (37.08)	0.00
Duration of PPI use (n, %)			0.22			0.37
Non-user	3434 (13.21)	4677 (18.00)		2377 (10.05)	4392 (18.57)	
<30 days	2979 (11.47)	4228 (16.27)		2790 (11.80)	4558 (19.27)	
30 to 180 days	5747 (22.12)	5081 (19.56)		5454 (23.06)	4855 (20.53)	
≥180 days	13,824 (53.20)	11,998 (46.17)		13,029 (55.09)	9845 (41.63)	

Abbreviations: IHD, Ischemic heart disease; CCI, Charlson Comorbidity Index; SBP, Systolic blood pressure; DBP, Diastolic blood pressure; FBG. Fasting blood glucose; PS, Propensity score; GERD, Gastroesophageal reflux disease. ^†^ Obesity (BMI, body mass index, kg/m^2^) was categorized as <18.5 (underweight), ≥18.5 to <23 (normal), ≥23 to <25 (overweight), ≥25 to <30 (obese I), and ≥30 (obese II).

**Table 2:** Crude and overlap propensity score-weighted odd ratios of proton pump inhibitor (ref: non-user) for stroke.

Characteristics	N of Stroke	N of Control I	Odd Ratios for Stroke (95% Confidence Interval)			
	(Exposure/Total, %)	(Exposure/Total, %)	Crude	*p*-Value	Overlap-Weighted Model ^†^	*p*-Value
Duration of PPI use						
Non-user	5197/42,048 (12.4)	16,893/84,096 (20.1)	1		1	
<30 days	4619/42,048 (11.0)	14,852/84,096 (17.7)	1.01 (0.97–1.06)	0.639	0.96 (0.92–1.00)	0.031 *
30 to 180 days	9052/42,048 (21.5)	17,001/84,096 (20.2)	1.73 (1.66–1.80)	<0.001 *	1.55 (1.50–1.61)	<0.001 *
≥180 days	23,180/42,048 (55.1)	35,350/84,096 (42.0)	2.13 (2.06–2.21)	<0.001 *	1.62 (1.57–1.68)	<0.001 *

* Significance at *p* < 0.05. ^†^ Adjusted for age, sex, income, region of residence, SBP, DBP, FBG, total cholesterol, obesity, smoking, alcohol consumption, CCI scores, GERD, and other forms of heart disease.

**Table 3:** Crude and overlap propensity score-weighted odd ratios of proton pump inhibitor (ref: non-user) for IHD.

Characteristics	N of IHD	N of Control II	Odd Ratios for IHD (95% Confidence Interval)			
	(Exposure/Total, %)	(Exposure/Total, %)	Crude	*p*-Value	Overlap-Weighted Model ^†^	*p*-Value
Duration of PPI use						
Non-user	3795/40,928 (9.3)	16,917/81,856 (20.7)	1		1	
<30 days	4551/40,928 (11.1)	17,202/81,856 (21.0)	1.18 (1.12–1.24)	<0.001 *	1.13 (1.08–1.18)	<0.001 *
30 to 180 days	9157/40,928 (22.4)	17,124/81,856 (20.9)	2.38 (2.28–2.49)	<0.001 *	2.12 (2.04–2.21)	<0.001 *
≥180 days	23,425/40,928 (57.2)	30,613/81,856 (37.4	3.41 (3.28–3.55)	<0.001 *	2.60 (2.51–2.69)	<0.001 *

* Significance at *p* < 0.05. ^†^ Adjusted for age, sex, income, region of residence, SBP, DBP, FBG, total cholesterol, obesity, smoking, alcohol consumption, CCI scores, GERD, and other forms of heart disease.

## Data Availability

Data are contained within the article and Supplementary Material.

## Supplementary Materials

The following supporting information can be downloaded at: https://www.mdpi.com/article/10.3390/biomedicines12010170/s1, Table S1: General characteristics of participants before propensity score overlap weighting adjustment.
