# Supplementary material for: Association between the Use of Proton Pump Inhibitors and Cardiovascular Diseases: A Nested Case-Control Study Using a National Health Screening Cohort
Source: Biomedicines. 2024 Jan 12;12(1):170. doi: 10.3390/biomedicines12010170 (PMC10813767; doi:10.3390/biomedicines12010170)
Supplement: Supplementary file 1 [file biomedicines-12-00170-s001.zip › biomedicines-2782854-supplementary.pdf]

Table S1. General Characteristics of Participants before propensity score overlap weighting adjustment

Characteristics	Before PS Overlap weighting adjustment			Before PS Overlap weighting adjustment		
	Stroke	Control I	Standardized Difference	IHD	Control II	Standardized Difference
Age (%)			0.00			0.00
40-44	70 (0.17)	140 (0.17)		250 (0.61)	500 (0.61)	
45-49	906 (2.15)	1,812 (2.15)		1,523 (3.72)	3,046 (3.72)	
50-54	2,650 (6.30)	5,300 (6.30)		3,423 (8.36)	6,846 (8.36)	
55-59	5,033 (11.97)	10,066 (11.97)		5,883 (14.37)	11,766 (14.37)	
60-64	5,907 (14.05)	11,814 (14.05)		6,737 (16.46)	13,474 (16.46)	
65-69	6,873 (16.35)	13,746 (16.35)		7,131 (17.42)	14,262 (17.42)	
70-74	7,913 (18.82)	15,826 (18.82)		6,579 (16.07)	13,158 (16.07)	
75-79	7,850 (18.67)	15,700 (18.67)		5,153 (12.59)	10,306 (12.59)	
80-84	4,511 (10.73)	9,022 (10.73)		2,965 (7.24)	5,930 (7.24)	
85+	335 (0.80)	670 (0.80)		1,284 (3.14)	2,568 (3.14)	
Sex (%)			0.00			0.00

Male	22,702 (53.99)	45,404 (53.99)		25,108 (61.35)	50,216 (61.35)	
Female	19,346 (46.01)	38,692 (46.01)		15,820 (38.65)	31,640 (38.65)	
Income (%)			0.00			0.00
1 (lowest)	7,664 (18.23)	15,328 (18.23)		7,009 (17.13)	14,018 (17.13)	
2	5,211 (12.39)	10,422 (12.39)		4,948 (12.09)	9,896 (12.09)	
3	6,432 (15.30)	12,864 (15.30)		6,105 (14.92)	12,210 (14.92)	
4	8,854 (21.06)	17,708 (21.06)		8,514 (20.80)	17,028 (20.80)	
5 (highest)	13,887 (33.03)	27,774 (33.03)		14,352 (35.07)	28,704 (35.07)	
Region of residence (%)			0.00			0.00
Urban	16,111 (38.32)	32,222 (38.32)		17,103 (41.79)	34,206 (41.79)	
Rural	25,937 (61.68)	51,874 (61.68)		23,825 (58.21)	47,650 (58.21)	
Obesity † (%)			0.11			0.22
Underweight	1,370 (3.26)	2,996 (3.56)		1,016 (2.48)	2,789 (3.41)	
Normal	14,222 (33.82)	30,927 (36.78)		11,938 (29.17)	29,933 (36.57)	
Overweight	11,073 (26.33)	22,211 (26.41)		10,990 (26.85)	22,156 (27.07)	

Obese I	13,898 (33.05)	25,629 (30.48)		15,331 (37.46)	24,753 (30.24)	
Obese II	1,485 (3.53)	2,333 (2.77)		1,653 (4.04)	2,225 (2.72)	
Smoking status (%)			0.08			0.07
Non-smoker	28,111 (66.85)	58,401 (69.45)		25,571 (62.48)	53,420 (65.26)	
Past smoker	3,349 (7.96)	7,311 (8.69)		4,040 (9.87)	8,080 (9.87)	
Current smoker	10,588 (25.18)	18,384 (21.86)		11,317 (27.65)	20,356 (24.87)	
Alcohol consumption (%)			0.04			0.06
<1 time a week	30,516 (72.57)	62,477 (74.29)		29,913 (73.09)	57,780 (70.59)	
≥1 time a week	11,532 (27.43)	21,619 (25.71)		11,015 (26.91)	24,076 (29.41)	
SBP (Mean, SD)	132.34 (18.03)	128.42 (16.62)	0.23	130.87 (17.37)	128.56 (16.80)	0.14
DBP (Mean, SD)	80.21 (11.27)	78.08 (10.41)	0.20	79.48 (10.93)	78.66 (10.57)	0.08
FBG (Mean, SD)	108.50 (38.44)	102.77 (29.21)	0.17	108.76 (39.02)	102.63 (29.97)	0.18
Total cholesterol (Mean, SD)	198.36 (40.90)	196.52 (39.12)	0.05	200.38 (42.21)	197.12 (37.98)	0.08
CCI score (Mean, SD)	1.68 (1.91)	0.91 (1.63)	0.43	1.35 (1.80)	0.98 (1.68)	0.21
GERD for 1 year before index date (Mean, SD)	0.68 (2.28)	0.53 (1.93)	0.07	0.82 (2.44)	0.44 (1.84)	0.18

Other forms of heart disease (n, %)	15,303 (36.39)	19,559 (23.26)	0.29	20,869 (50.99)	16,053 (19.61)	0.70
Duration of PPI use (n, %)			0.34			0.52
Non-user	5,197 (12.36)	16,893 (20.09)		3,795 (9.27)	16,917 (20.67)	
< 30 days	4,619 (10.99)	14,852 (17.66)		4,551 (11.12)	17,202 (21.01)	
30 to 180 days	9,052 (21.53)	17,001 (20.22)		9,157 (22.37)	17,124 (20.92)	
≥ 180 days	23,180 (55.13)	35,350 (42.04)		23,425 (57.23)	30,613 (37.40)	

Abbreviations: CCI, Charlson Comorbidity Index; SBP, Systolic blood pressure; DBP, Diastolic blood pressure; FBG. Fasting blood glucose; PS, Propensity score; GERD, Gastroesophageal reflux disease;

† Obesity (BMI, body mass index, kg/m^2) was categorized as < 18.5 (underweight), ≥ 18.5 to < 23 (normal), ≥ 23 to < 25 (overweight), ≥ 25 to < 30 (obese I), and ≥ 30 (obese II)
